# A case report combining Dunbar syndrome and pancreatic neuroendocrine tumor

**DOI:** 10.1016/j.amsu.2020.08.038

**Published:** 2020-09-01

**Authors:** Fatema Alzahraa Almohamad, Maryam Alhimyar, Rami Esmaeel, Bayan Alsaid

**Affiliations:** a Department of General Surgery, Al-Assad University Hospital, Damascus, Syria; b Faculty of Medicine, Damascus University, Damascus, Syria; c Laboratory of Anatomy, Faculty of Medicine, Damascus University, Damascus, Syria

**Keywords:** Dunbar syndrome, Median arcuate ligament syndrome, Celiac trunk, Pancreatic neuroendocrine tumors, CK, focal cytokeratins, DS, Dunbar syndrome, EUS, endoscopic ultrasonography, IHC, Immunohistochemistry, MALS, median arcuate ligament syndrome, MsCT, Multi slice computer tomography, MRA, magnetic resonance angiography, MRI, magnetic resonance imaging, NSE, neuron specific enolase, PNETs, pancreatic neuroendocrine tumors, SRS, somatostatin receptor scintigraphy

## Abstract

**Background:**

Dunbar syndrome or median arcuate ligament syndrome is a rare disorder. In this disorder, a malposition of the arcuate ligament compresses the celiac trunk and causes nonspecific symptoms including postprandial pain, abdominal bruit and weight loss. Surgical management is the primary treatment.

Pancreatic neuroendocrine tumors (PNETS) are also rare. It comprises about 1–3% of pancreatic neoplasm. The patient could be symptomatic or asymptomatic depends on the tumor being functional or nonfunctional. In addition, surgical therapy is the choice.

**Case presentation:**

In this paper, we report a case of 28 old female patient complaining from a long term of recurrent abdominal pain that doesn't releive on any kind of treatment, the multislices computerised tomography scan showed compress of the median arcuate ligament with an incidental mass in the tail of pancreas turned out to be a pancreatic neuroendocrine tumor.

**Conclusions:**

The Dunbar syndrome and the Pancreatic neuroendocrine tumors must be kept in mind of phyciciens while the differential diagnosis of any recurrent abdominal pain.

## Highlights

• Dunbar syndrome or median arcuate ligament syndrome is a rare disorder which presented with abdominal pain.
• Celiac artery compression can be diagnosed with Multi slices computer tomography and selective catheter angiography, surgery is the treatment of choice.
• The Dunbar syndrome and the Pancreatic neuroendocrine tumors must be kept in mind during differential diagnosis of recurrent abdominal pain.

## 1 Introduction

Median arcuate ligament syndrome (MALS) or Dunbar syndrome (DS) is a rare disorder with nonspecific symptoms.

The median arcuate ligament (MAL) is a fibrous ligament connecting the right and left diaphragmatic crura, rises at the level of 12th thoracic vertebra and 1st lumbar vertebra [1]. In Dunbar syndrome, malposition of the MAL causes a compression on the celiac trunk and leading to the symptoms of abdominal angina. MALS or celiac artery compression can be diagnosed with Doppler ultrasound, Multi slices computer tomography, selective catheter angiography, and magnetic resonance angiography (MRA) [2]. Surgery is the treatment of choice, cutting fibers of the mal ligament by either open or laparoscopic approach (3).

Pancreatic neuroendocrine tumors (PNETs) are also rare. It comprises only about 1–3% of pancreatic neoplasms [4]. PNETs can be associated with other conditions like multiple neoplasia type 1 (MEN1), Von Hippel Lindau syndrome and neurofibromatosis Type 1 [5]. PNETs can be silent or symptomatic; the symptoms could be a result of invasion or hormone secretion. Symptoms can be varied from abdominal pain, weight loss to the signs of hypoglycemia, hypokalemia or diabetes. Depending on which kind of hormone they are secreting [6]. Nonfunctional tumors comprises about 15%–53% and most commonly located in the head of pancreas [7], these tumors could be detected incidentally on imaging before developing symptoms [8], The treatment of localized neuroendocrine tumors is surgical resection [6].

Some studies report the coexistence of Dunbar syndrome with pancreatic carcinoma [[9], [10], [11], [12], [13]] with present a surgical challenge during the resection of the pancreatic head. To our knowledge, silent PNETs was not reported in combination of Dunbar syndrome. Here we present a 28-year old female with a history of abdominal pain, diagnosed with Dunbar syndrome and neuroendocrine tumor in the tail of pancreas. This work has been reported in line with the SCARE criteria [14].

### 1.1 Case presentation

A 28-year old female presented in our center suffering from a long term of recurrent abdominal pain. The chronic recurrent postprandial pain started six years ago.

The daily pain localized in the epigastric region, the pain wakes her up in the night and she had no complete relieve on analgesics. No nausea, vomiting or weight loss were recorded. Moreover, no diarrhea or constipation were detected. No previous history for medical or family conditions. Only She had an appendectomy ten years ago and two cesareans, and cholecystectomy five years ago.

Three upper endoscopies showed normal findings with almost six months between one and another. She has a previous diagnosis of irritable bowel syndrome to explain her recurrent abdominal pain.

**Fig. 1:**
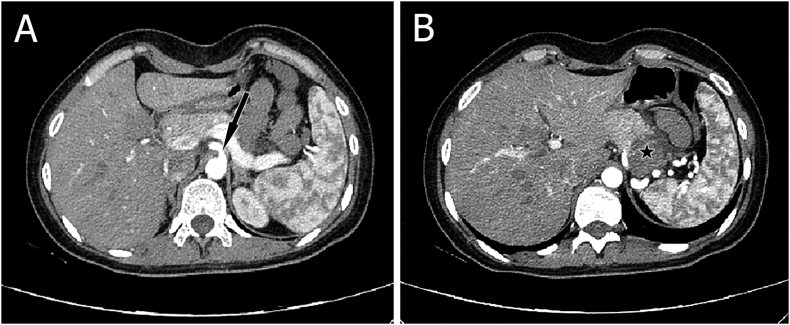
**A-** Celiac trunk angulation (arrow). **B-** The mass in the tail of pancreas (star).

At admission, abdominal examination, laboratory tests and ultrasound were within normal limits. Multi slice computed tomography (MsCT) revealed a 3.5 cm heterogeneous mass in the tail of pancreas with stenosis up to 40% in the celiac trunk with 45° of angulation (Fig. 1).

**Fig. 2:**
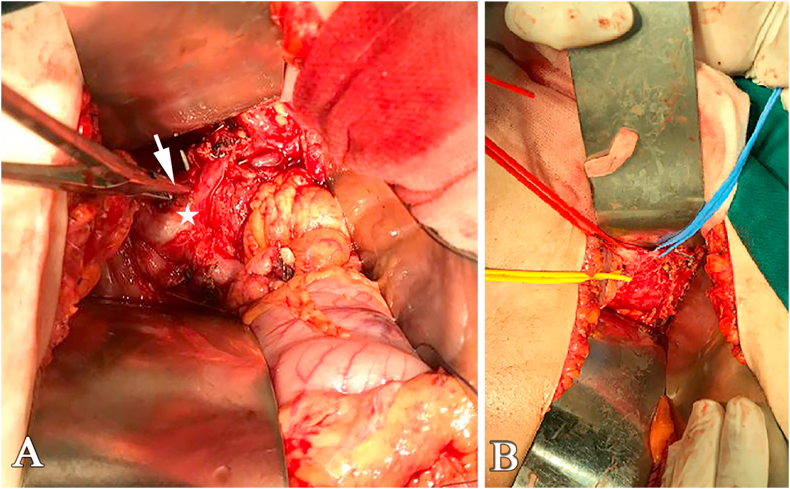
**A-** The arrow referred to the arcuate ligament and the star to the celiac trunk. **B-** Celiac trunk and its branches: hepatic artery (red band), left gastric artery (blue band) and splendid artery (yellow band). (For interpretation of the references to colour in this figure legend, the reader is referred to the Web version of this article.)

The diagnosed of DS was probable, and a surgical intervention was proposed to resolve the celiac trunk compression and to resect the pancreatic tumor. Abdominal exploration was performed via midline incision; a 3.5 cm mass in the tail of the pancreas was detected with no metastasis or ascites. Distal pancreatectomy with splenectomy was performed. Liberation of the celiac trunk was accomplished by cutting the median arcuate ligament (Fig. 2).

The patients discharged from the hospital three days after surgery in good situation. Follow up after showed a complete relieve for her pain.

The pathology result showed neuroendocrine tumor in the tail of pancreas.

A well-demarcated mass in the pancreatic tail, measures 3cm in the greatest dimension, composed of proliferation of sheets and nests of medium and large cells with eosinophilic and finely granular cytoplasm, nuclei are round with salt and pepper chromatin. Atypical and bizarre cell present with prominent mitotic figures and tumor necrosis. Rich vascular network in the stroma, with fibrotic areas.

**Fig. 3:**
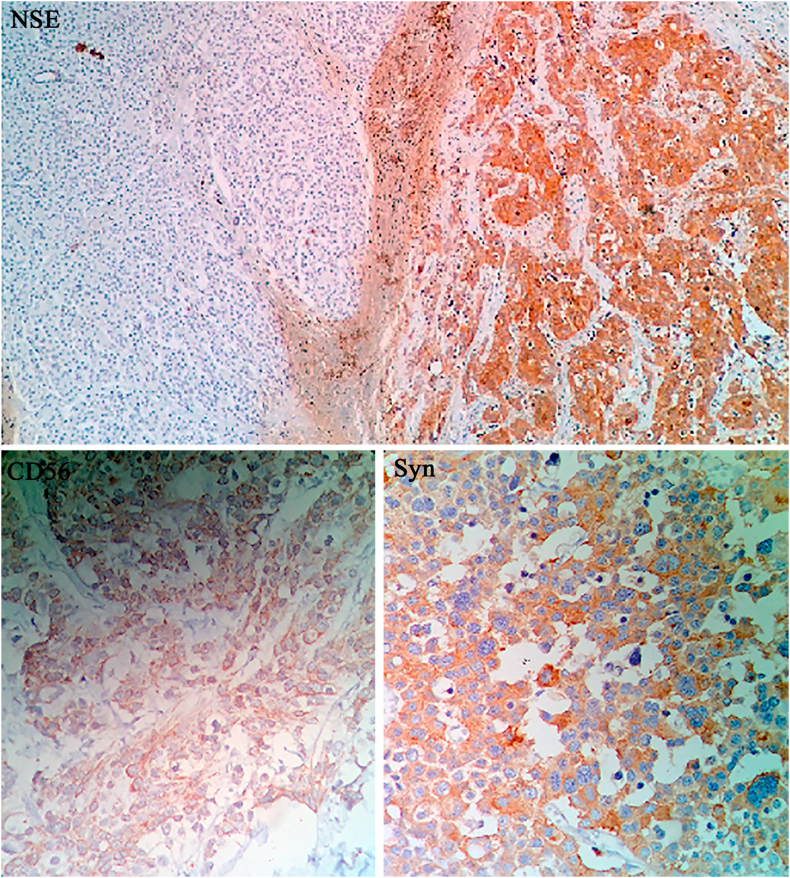
Immunohistochemistry (IHC) showing positivity to neuron specific enolase (NSE), CD56 and synaptophysin (Syn).

Immunohistochemistry (IHC) revealed that neuron specific enolase (NSE), synaptophysin and CD56 were positive (diffuse), negative chromogranin, focal cytokeratins (CK) and the KI67 was high index (20%). Finally, Diagnosis is a well-differentiated neuroendocrine tumor, Grade 3 (Fig. 3).

## 2 Discussion

MALS or called Dunbar syndrome is a rare reason for abdominal pain with nonspecific symptoms. It was first described in 1963 by Harjola et al. [1,15].

Symptoms can be explained by two theories, the first is the malposition of the median arcuate ligament, which compresses the celiac trunk and causes mesenteric ischemia. The second is the direct irritation of the celiac ganglion or compression of the ganglion plexus lead to painful neuro stimulation [1,3].

Nonspecific symptoms and vague pain makes delay the diagnose [3].

MALS or celiac artery compression can be diagnosed with Doppler ultrasound, Multi slices computer tomography, selective catheter angiography, and magnetic resonance angiography (MRA) [2]. Pre- and postsurgical peak systolic velocity was recommended to confirm the diagnosis of this controversial syndrome.

In our case the our diagnosis was made by computed tomography angiography (CTA), and no collateral circulation was observed, the patients with symptomatic celiac axis stenosis often have multiple vessel obstruction or severe stenosis. When the collateral circulation is poor, that is Gastroduodenal artery hypoplasia, the patients with single celiac axis stenosis cloud have postprandial abdominal pain.

Surgical decompression of the celiac trunk and celiac plexuses is the treatment of choice, it can be done by either open or laparoscopic approach [3].

Neuroendocrine tumors are also rare tumors, which can be located in different organs as gastrointestinal tract, pancreas and ovaries [16].

Pancreatic neuroendocrine tumors (PNETs) comprises about 1–3% of all pancreatic neoplasms [4].

PNETs can be silent or nonfunctional, contrarily to insulinomas, glucagonomas, VIPomas, and somatostatinomas, and other rare types, which caused symptoms according to the hormone secretion [7].

PNETs can be detected by computer tomography (CT), magnetic resonance imaging (MRI), endoscopic ultrasonography (EUS), and somatostatin receptor scintigraphy (SRS) which is recommended in most guidelines [7].

Surgical therapy for Dunbar syndrome or MALS can be varied from transaction of median arcuate ligament, destruction of the celiac ganglion or revascularization of the decompressed celiac truck [1].

In our patient the pain was chronic and recurrent daily non-relieving despite of all treatments. Surgical decompression of the median arcuate ligament relieved her symptoms after surgery.

Moreover, our patient had a neuroendocrine tumor in the tail of pancreas, which makes the question could her pain caused by the mass and MALS syndrome discovered incidentally or the pain caused by MALS and the mass detected incidentally.

There is an increasing number of incidentally discovered PNETs on abdominal imaging performed for other reasons [6].

PENTs can be associated with other conditions like Multiple Neoplasia Type 1 (MEN1), Von Hippel Lindau syndrome and Neurofibromatosis Type 1 [7].

Yasuo Shima et al. and Jen-Wei Chou et al. described tumors in the head of pancreas with MALS. However, no other articles documented MALS with neoplasm in the tail of pancreas [17,18].

To our knowledge, this is the first documented MALS syndrome with neoplasm of tail of pancreas.

For locoregional PNETs, surgical resection is the treatment. Functional PENTs give symptoms and help to discover the tumor in early stages.

On the other hand, nonfunctional tumors are being locally advanced when it has been diagnosed.

Most PENTs are malignant except for insulinomas, and oncologic principles should be followed during the resection [6].

Our patient had incidentally discovered the mass during the MSCT performed to detect any compression on the celiac artery, And with one surgery relieved her pain by removing the compression on celiac artery and distal Pancreatectomy with splenectomy accomplished to remove the mass.

## 3 Conclusion

Although MALS are rare, we should keep in mind Dunbar syndrome or MALS as a differential diagnosis in any patient with abdominal recurrent pain.

As to PNETs still incidentally detected and mysterious as long they are nonfunctional tumors.

## 4 Ethical Approval and consent to participate

Ethical Approval upon data access and consent to participate was given from the directory of surgical department in Al-assad university hospital.

## 5 Consent for publication

Written informed consent was obtained from the patient for publication of this case report and any accompanying images.

## 6 Availability of supporting data

All data are available.

## Competing interests

The authors declare that they have no competing interests.

## Funding

No funding was received in support of this work.

## Authors' contributions

The operation was done by RE and BA. The literature search was done by FA and MA. FA, MA, and BA wrote the manuscript. RE revised the manuscript.

## Provenance and peer review

Not commissioned, externally peer reviewed.

## Declaration of competing interest

The authors declare that there are no conflicts of interest regarding the publication of this paper.
